# Transseptal Puncture Through an Interatrial Septum With Lipomatous Hypertrophy: A False Perception of Success and Failure

**DOI:** 10.7759/cureus.29737

**Published:** 2022-09-29

**Authors:** Intisar Ahmed, Aman Qureshi, Aamir H Khan

**Affiliations:** 1 Cardiology, Department of Medicine, Aga Khan University, Karachi, PAK; 2 Department of Medicine, Aga Khan University, Karachi, PAK

**Keywords:** dissection, lipomatous hypertrophy, interatrial septum, puncture, transseptal

## Abstract

Transseptal puncture is a standard procedure to access the left atrium during catheter ablation of arrhythmias. It is associated with high success and a meager complication rate in the contemporary era. The potential complications of transseptal puncture include aortic puncture, perforation of the right/left atrial free wall resulting in pericardial effusion/tamponade, and systemic thromboembolism. The dissection of the interatrial septum (IAS) is a rare complication of transseptal puncture, reported in less than 1% of the procedures. We report a case of a dissection of the IAS during an attempted transseptal puncture in a 72-year-old man with lipomatous hypertrophy of the interatrial septum.

## Introduction

Atrial fibrillation (AF) is the most common tachyarrhythmia, especially in the elderly population, and is a major contributor to cerebrovascular accidents [1]. AF catheter ablation is recommended in a select group of highly symptomatic patients who do not tolerate antiarrhythmic drugs [2]. Access to the left atrium is required for AF catheter ablation, which is usually obtained via transseptal puncture. The transseptal approach and direct measurement of left atrial pressure were first described by Cournand in the 1940s in children with interatrial septal defects [3]. Brockenbrough and colleagues introduced the modern transseptal puncture technique in 1962 [4]. Since then, transseptal puncture has been widely used in catheter ablation of arrhythmias as well as percutaneous intervention in structural heart diseases. The transseptal puncture has a high success and a low complication rate and most of the complications are related to interatrial septal anatomy and right and left atrial dimensions [5]. Interatrial septal dissection is a rare complication of transseptal puncture, reported in 0.5% of the cases [6].

Here, we report a case of dissection of the interatrial septum (IAS) during an attempted transseptal puncture in a patient with lipomatous hypertrophy of the interatrial septum (LHIS).

## Case presentation

**Figure 1:**
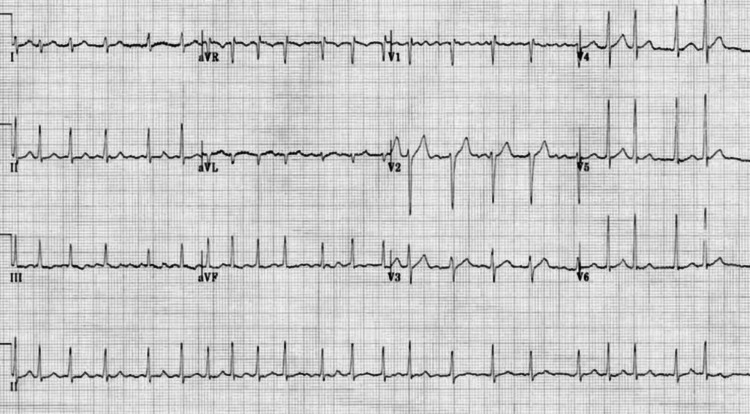
Twelve-lead electrocardiogram revealing atrial fibrillation with rapid ventricular rate.

A 72-year-old caucasian man with a history of hypertension presented with palpitations and near syncope. He was found to have paroxysmal AF and prolonged offset pauses upon conversion to sinus rhythm. He had been treated with sotalol and diltiazem but remained symptomatic. Physical examination revealed hypertension and was otherwise normal. His 12-lead electrocardiogram (ECG) demonstrated AF with a ventricular rate of around 130 beats per minute (Figure 1).

**Figure 2:**
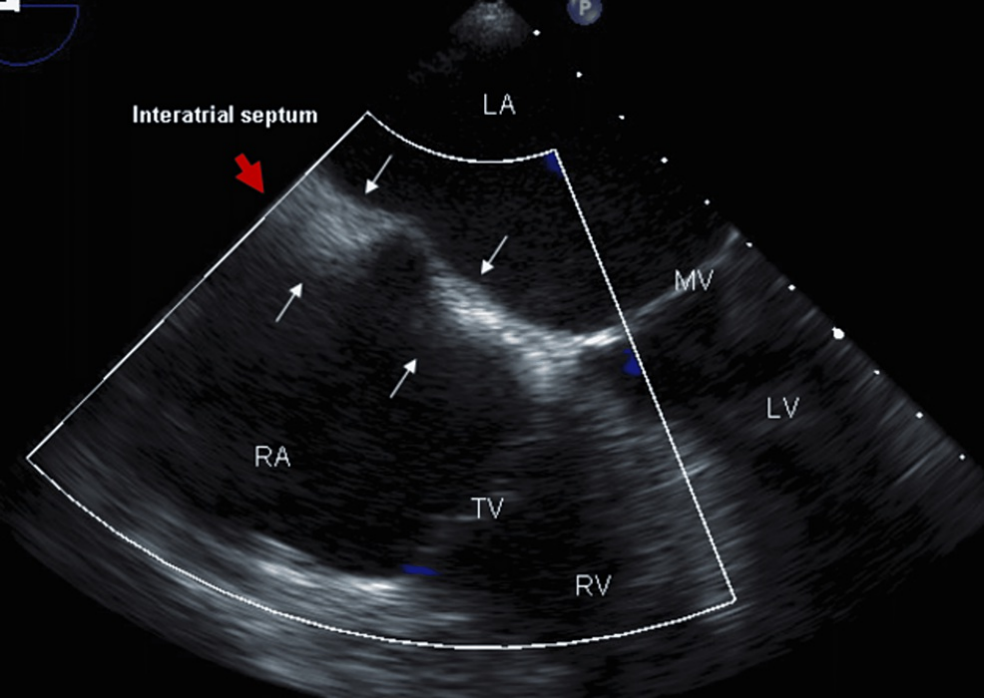
Transesophageal echocardiogram revealing thickened interatrial septum with sparing of fossa ovalis, consistent with lipomatous hypertrophy of interatrial septum. LA: left atrium; RA: left atrium; LV: left ventricle; RV: right ventricle; TV: tricuspid valve, MV: mitral valve

Before ablation, he underwent transesophageal echocardiography (TEE), which revealed normal left ventricular systolic function and no left atrial appendage thrombus. The interatrial septum was noted to be thick with sparing of fossa ovalis, with a characteristic dumbbell shape suggestive of lipomatous hypertrophy (Figure 2).

**Figure 3:**
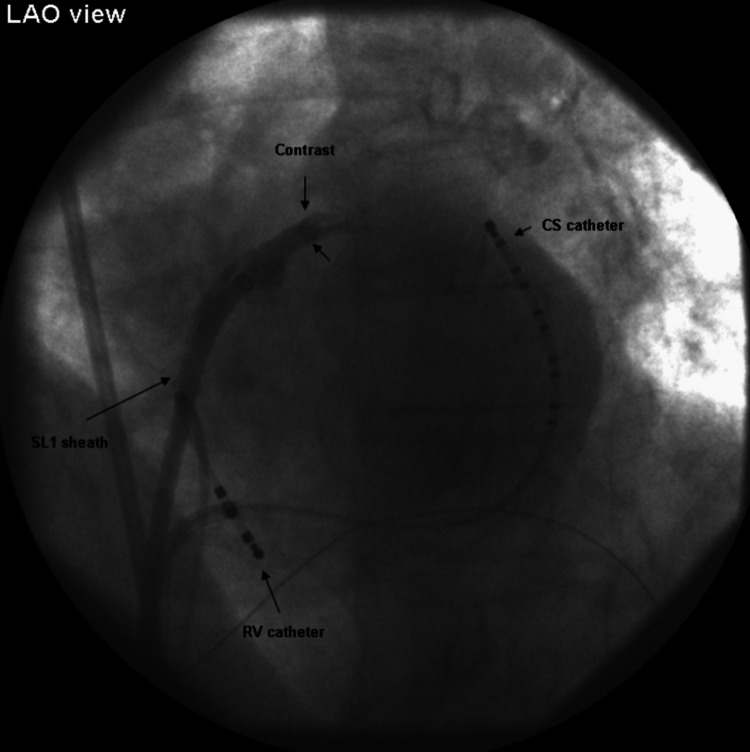
Cine angiography. Contrast injection via SL1 sheath shows contrast enhancement of the roof with no extravasation in the pericardium. CS: coronary sinus; RV: right ventricle

All four pulmonary veins were visualized and appeared normal. A computed tomography (CT) scan of the left atrium and pulmonary veins demonstrated variant pulmonary vein anatomy with a right middle lobe pulmonary vein. In the electrophysiology (EP) laboratory, femoral venous access was obtained during light conscious sedation, and electrode catheters were placed in the coronary sinus and the right ventricular apex. Fluoroscopy, with a posteroanterior view of the image intensifier, was used, and the sheath was cannulated with a Brockenbrough needle through which continuous pressure monitoring was performed. An 8.5-French SL1 sheath (Abbott Cardiovascular) was utilized to attempt transseptal puncture. During pull back from the superior vena cava, the sheath tip appeared to drop below the root of the aorta and was then positioned posteriorly, engaging a structure believed to be the fossa ovalis on visual and tactile evaluation. Upon advancement of the needle, a pop was felt and the sheath tip advanced easily. The pressure recording was suggestive of left atrial pressure, but blood could not be aspirated from the needle. The needle was thus withdrawn and a volume of radiocontrast was injected. Cine angiography (with left anterior oblique angulation), was highly suggestive of dissection of the interatrial septum without penetration of the pericardial space (Figure 3).

The patient remained asymptomatic. The sheath was withdrawn, and a rotational intracardiac echocardiography probe (Ultra-ICE, Bo-Sci) was advanced via another sheath. The foramen ovale was identified, and a repeat puncture attempt was performed during visualization of the septum to confirm penetration of the left atrium prior to sheath advancement. Heparin 2,000 units were administered via the sheath, and a slow infusion of heparinized saline was initiated and maintained. This was followed by a second transseptal puncture. Pulmonary vein isolation was performed uneventfully during which intermittent bolus heparin was administered to maintain the activated clotting time greater than 300 seconds. The patient was discharged the following day. He suffered an early recurrence of AF and a repeat procedure (again using intracardiac echocardiography for the transseptal puncture) was performed. He remained asymptomatic on follow-up.

## Discussion

LHIS was first described in 1964 [7]. It is a rare entity that is characterized by excessive deposition of fat in the interatrial septum, with a thickness of greater than 2 cm, and is more frequently observed in the elderly [8]. Although usually asymptomatic, it has been associated with supraventricular tachyarrhythmias and sudden death [9]. In this case, we thought it was likely responsible for the dissection of the interatrial septum during what appeared to be a routine transseptal puncture. The sheath was fortunately not advanced due to the inability to withdraw blood through the needle. Although the hypertrophied interatrial septum was visible on the TEE examination and CT prior to the procedure, its clinical importance was not recognized. This case highlights the role of multimodality imaging in the evaluation of cardiac anatomy in patients undergoing electrophysiology procedures.

Lipomatous hypertrophy consists of an unencapsulated accumulation of mature adipose tissue with cells resembling brown fat which demonstrates vacuolated cytoplasm. It is caused by the proliferation of fat cells and not the hypertrophy of cells [10]. The cephalad portion of the septum is usually thicker than the caudal portion and the fossa is usually spared [8]. It is generally confined to the atrium. In one study, the masses were noted to project only into the right atrium, although extension into the left atrium free wall has also been noted [11,12]. The first non-operative report of diagnosis in a living human was through a CT scan in 1982, but currently, transthoracic echocardiogram, TEE, or CT/MRI scan are recommended [13,14].

## Conclusions

Transseptal puncture is increasingly performed for interventional procedures, prior to which imaging studies are frequently performed. Awareness of the potential for lipomatous hypertrophy of the IAS to contribute to dissection or possibly perforation may increase its recognition in pre-procedure imaging. Direct imaging of the interatrial septum during transseptal puncture using intracardiac echocardiography or TEE may reduce the risk of complications associated with transseptal puncture.
